# The effect of polymyxin B hemoperfusion on modulation of human leukocyte antigen DR in severe sepsis patients

**DOI:** 10.1186/s13054-018-2077-y

**Published:** 2018-10-26

**Authors:** Nattachai Srisawat, Somkanya Tungsanga, Nuttha Lumlertgul, Chalermchai Komaenthammasophon, Sadudee Peerapornratana, Nicha Thamrongsat, Khajohn Tiranathanagul, Kearkiat Praditpornsilpa, Somchai Eiam-Ong, Kriang Tungsanga, John A. Kellum

**Affiliations:** 1 Division of Nephrology, Department of Medicine, Faculty of Medicine, Chulalongkorn University, Bangkok, 10330 Thailand; 2 Excellence Center for Critical Care Nephrology, King Chulalongkorn Memorial Hospital, Thai Red Cross Society, Bangkok, Thailand; 3 Department of Critical Care Medicine, The Center for Critical Care Nephrology, CRISMA, University of Pittsburgh School of Medicine, Pittsburgh, PA USA

**Keywords:** Immunoparalysis, Severe sepsis/septic shock, Polymyxin B hemoperfusion

## Abstract

**Background:**

Recent randomized trials have not found that polymyxin B hemoperfusion (PMX-HP) improves outcomes for patients with sepsis. However, it remains unclear whether the therapy could provide benefit for highly selected patients. Monocyte human leukocyte antigen (mHLA-DR) expression, a critical step in the immune response, is decreased during sepsis and leads to worsening sepsis outcomes. One recent study found that PMX-HP increased mHLA-DR expression while another found that the treatment removed HLA-DR-positive cells.

**Methods:**

We conducted a randomized controlled trial in patients with blood endotoxin activity assay (EAA) level ≥ 0.6. Patients in the PMX-HP group received a 2-h PMX-HP treatment plus standard treatment for 2 consecutive days. Patients in the non-PMX-HP group received only standard treatment. The primary outcome compared the groups on median change in mHLA-DR expression between day 3 and baseline. Secondary outcomes compared the groups on the mean or median change in CD11b expression, neutrophil chemotaxis, presepsin, cardiovascular Sequential Organ Failure Assessment (CVS SOFA) score, vasopressor dose, and EAA level between day 3 and baseline. We further compared the groups on mortality, ICU-free days, ventilator-free days, dialysis dependence status, renal recovery, serum creatinine, vasopressor-free days, and major adverse kidney events (MAKE 28), measured on day 28.

**Results:**

Fifty-nine patients were randomized to PMX-HP (*n* = 29) and non-PMX-HP (*n* = 30) groups. At baseline, mHLA-DR expression, CD11b, neutrophil chemotaxis, and clinical parameters were comparable between groups. The median change in mHLA-DR expression between day 3 and baseline was higher in PMX-HP patients than in patients receiving standard therapy alone (*P* = 0.027). The mean change in CD11b between day 3 and baseline was significantly lower in the PMX-HP group than in the non-PMX-HP group (*P* = 0.002). There were no significant changes from baseline in neutrophil chemotaxis, presepsin, CVS SOFA scores, vasopressor doses, or EAA level between groups. On day 28 after enrollment, mortality, ICU-free days, ventilator-free days, dialysis dependence status, renal recovery, serum creatinine, vasopressor-free days, and MAKE 28 were comparable between groups.

**Conclusion:**

PMX-HP improved mHLA-DR expression in severe sepsis patients. Future studies should examine the potential benefit of PMX-HP in patients with low mHLA-DR expression.

**Trial registration:**

ClinicalTrials.gov, NCT02413541. Registered on 3 March 2015.

**Electronic supplementary material:**

The online version of this article (10.1186/s13054-018-2077-y) contains supplementary material, which is available to authorized users.

## Background

Although there has been substantial improvement of care through the use of potent antibiotics and sepsis care bundles, severe sepsis/septic shock is still the leading cause of death worldwide [1–3].

For more than 25 years, polymyxin B hemoperfusion (PMX-HP) has been introduced as one of the treatments for sepsis in Japan, and recently in Europe and Asia [4, 5]. It was reported that polymyxin B could neutralize the various biologic activities of endotoxins [6]. Data from a noncontrolled clinical study demonstrated that PMX-HP might improve sepsis outcomes by diminishing the negative effects of hypotension [7], reducing serum cytokine levels [8] or improving monocyte mRNA expression [9, 10]. However, the molecular mechanism of PMX-HP and its usefulness is not yet clear and has never been tested in a randomized controlled trial.

During the early phase of sepsis there is a massive release of proinflammatory cytokines which are responsible for organ dysfunction and tissue hypoperfusion. Simultaneously there is an anti-inflammatory response to balance the body’s homeostasis and to avoid the excessive effect of a proinflammatory response. However, this effect might lead to a state of immunoparalysis and cause secondary infections from opportunistic pathogens [11]. Unfortunately, we still lack an effective treatment to improve the immunoparalysis state.

The monocyte/macrophage system plays one of the major roles in host defense. The main functions of monocytes, antigen expression, and cytokine production are mediated by certain surface molecules from major histocompatibility complex (MHC) class II, such as monocyte human leukocyte antigen (mHLA-DR). mHLA-DR allows antigen presentation to T cells and is crucial for the initiation of the cascade of the immune response during sepsis [12]. Therefore, the diminished expression of mHLA-DR could represent a state of immunoparalysis and would lead to higher risk of secondary infection. A recent report also showed that low mHLA-DR expression might relate to higher mortality rates [13]. However, recent concepts have been put forth including “tolerance” which posit that suppression of the immune system can be beneficial for limiting tissue damage. Thus, it remains unclear whether to use drugs to boost the immune system [14].

We conducted a randomized controlled trial to evaluate the effect of PMX-HP on mHLA-DR expression. We also explored the effect of PMX-HP on CD11b expression on the neutrophil, neutrophil chemotaxis, cardiovascular Sequential Organ Failure Assessment (CVS SOFA) score, vasopressor dose, and endotoxin level. Furthermore, mortality, intensive care unit (ICU)-free days, ventilator-free days, dialysis dependence status, renal recovery, and serum creatinine were compared on day 28.

## Methods

The study was conducted at King Chulalongkorn Memorial Hospital, Bangkok, Thailand in five ICUs: two medical, one coronary care, and two surgical. The study was conducted between February 2014 and July 2017. The protocol was approved by the Faculty Ethical Committee (IRB No. 578/56) and registered at ClinicalTrials.gov (NCT02413541). Informed consent was obtained from each patient or the patient’s surrogate.

### Study population

Patients were eligible to participate in the trial if they were 18 years of age or older, and were diagnosed with severe sepsis or septic shock according to the consensus definition of the American College of Chest Physicians (ACCP)/Society of Critical Care Medicine (SCCM) Consensus Conference Committee [15] regardless of renal function or blood pressure. Patients were not eligible if they had one of the following: expected to die within 24 h, pregnant, white blood cell count less than 500/mm^3^, platelet count less than 30 × 10^3^ /mm^3^, uncontrolled coagulopathy, terminal illness with Do-Not-Resuscitate will, allergic reaction to PMX-HP, or organ transplantation.

### Randomization

On admission to the ICU venous blood was collected for endotoxin activity assay (EAA). Patients with EAA levels of 0.6 or greater were randomly assigned by blocked randomization into two groups: the PMX-HP group and the non-PMX-HP group. Although the allocation sequence was concealed it was not possible to conceal the intervention among the two groups. However, during the analysis the investigators were blinded to the group allocation of each subject.

### Study treatments

All patients were treated with the standard treatment according to the Surviving Sepsis Campaign guideline [16] by physicians with experience in extracorporeal blood purification techniques. Venous access was undergone using a double-lumen catheter. Patients in the PMX-HP group received a 2-h PMX-HP treatment plus standard treatment for 2 consecutive days after enrollment. The hemoperfusion was done with adsorbent columns consisting of 5 mg of polymyxin B per gram of polystyrene fiber (Toraymyxin^®^; Toray Industries, Tokyo, Japan). Access to blood for PMX-HP treatment was obtained through a double-lumen catheter inserted into the central venous by Seldinger’s method. The blood flow rate was set at 120 ml/min. Heparin infusion was the standard anticoagulant. If a patient had contraindication to heparin we used the nonanticoagulant strategy. Neither volume replacement nor ultrafiltration was conducted during the hemoperfusion process.

### Endotoxin activity assay

We used the chemiluminescent-based endotoxin activity assay (Spectral Diagnostics, Ontario, Canada) as described elsewhere [17]. This assay is based on the detection of enhanced respiratory burst activity in neutrophils following their priming by complexes of endotoxin and a specific anti-endotoxin antibody. Briefly, 40 μl of whole blood were incubated with zymosan and anti-endotoxin antibody. The immune complex between endotoxin and specific antibody potentiates the zymosan-driven reactive oxygen species (ROS) production by neutrophils. The amount of ROS released indicates the relative amount of endotoxin in blood [17–19]. ROS were determined by chemiluminescent assay normalized for the neutrophils’ ability to produce ROS.

### Measurement of mHLA-DR expression, CD11b expression on neutrophils, and neutrophil chemotaxis

#### Neutrophil isolation

Whole blood was collected by venipuncture into heparinized tubes. Polymorphonuclear cells (PMN) and mononuclear cells were extracted by one-step centrifugation with Polymorphprep™, which contained sodium diatrizoate and an 8% (w/v) polysaccharide solution (AxisShield, Norway). Fresh blood was layered onto PolymorphPrep™ in a 1:1 ratio and centrifuged for 30 min at 500 × *g* (1700–1800 rpm). This density gradient allows separation of both monocytes and PMN. Following separation, the monocyte and PMN layer was removed with a sterile pipette and washed with RPMI 1640 by centrifugation at 800 × *g* (2000 rpm) for 5 min to remove residual PolymorphPrep™. Contaminating erythrocytes were remove by resuspending the cell pellet in ammonium chloride lysis buffer in 9:1 ratio with RPMI 1640 for 3 min. Cells were pelleted at 800 × *g* for 5 min and resuspended in RPMI 1640 supplemented with 5–10% (v/v) human AB serum (or fetal bovine serum).

#### Flow cytometry for detecting mHLA-DR expression and CD11b expression on neutrophils

The CD11b expression on neutrophils and HLA-DR expression on monocytes were measured using monoclonal antibody staining and flow cytometry. Neutrophils and monocytes were collected and then incubated with allophycocyanin (APC)-conjugated CD11b (IgG1; Beckman Coulter, France) and with phycoerythrin (PE)-conjugated HLA-DR (IgG1; Beckman Coulter) for 30 min, respectively. After incubation the samples were washed twice in PBS without calcium or magnesium and fixed with 4% paraformaldehyde. All samples were run within 7 days of collection by a flow cytometer (BD LSR II; BD Biosciences, USA) using acquisition and analysis software (BD FACSDiva; BD Biosciences).

#### Neutrophil chemotaxis

The chemotaxis assay was performed using Hanging Millicell inserts (Merck Millipore, Germany). Twenty-four-well tissue culture well plates were coated with poly(2-hydroxyethyl methacrylate) to prevent cell adhesion following transmigration. Then 800 μl RPMI 1640 with chemoattractant (10^− 9^ mol/L formyl methionyl-phenylalanine (FMLP)) was added to each well. The hanging inserts with a 3-μm pore-size filter at their base and 1 × 10^6^ neutrophils in 200 μl culture media were added into the hanging inserts and incubated at 37 °C in a 5% CO_2_ incubator for 90 min. The hanging inserts were then removed and migrated neutrophils in each well were counted. The count number of migrated neutrophils was calculated as a percentage of the total number of cells originally added [20].

### Measurement of presepsin

Presepsin concentrations in plasma were measured using a compact fully automated immunoassay analyzer (PATHFASTTM System; LSI Medience Corporation, Japan/Mitsubishi Chemical Europe), based on a noncompetitive chemiluminescence enzyme immunoassay [21].

### Data collection

Clinical data and laboratory testing were collected on days 1, 2, 3, 7, 14, 21, and 28 after enrollment. mHLA-DR expression, CD11b expression, neutrophil chemotaxis, and EAA levels were assessed on days 1 and 3. Clinical severity was assessed on the aforementioned days using the SOFA score [22]. The amount of vasoactive agents used was measured by the inotropic score [23, 24] and the vasopressor index [25, 26].

### Outcomes

The primary outcome was the change in mHLA-DR expression, measured as mHLA-DR on day 3 after enrollment minus mHLA-DR at baseline. Secondary outcomes, defined as change between day 3 and baseline, included: CD11b expression on neutrophil, neutrophil chemotaxis, CVS SOFA score, vasopressor dose, and EAA level. Finally, clinical outcomes measured on day 28 included: mortality, ICU-free days, ventilator-free days, dialysis dependence status, renal recovery, serum creatinine, vasopressor-free day, and major adverse kidney events (MAKE) by day 28. Primary and secondary outcomes were measured on those alive on day 3. Outcomes on day 28 were measured in those still alive at the start of the day.

### Statistical analysis

According to Ono et al. [10], in order to detect a 15% difference in mHLA-DR expression between groups a sample size of 60 was needed: 30 patients for the PMX-HP group and 30 patients for the non-PMX-HP group.

Statistical analyses were performed using Stata version 15.0, with statistical significance set at *P* < 0.05. Comparisons between groups were performed with Fisher’s exact test for categorical variables and using Student’s *t* test or Kruskal–Wallis one-way analysis of variance by ranks for continuous variables. The main and secondary outcomes, calculated as change from baseline, were tested between groups with either Student’s *t* test or Kruskal–Wallis test depending on their distribution. Kaplan–Meier survival curves were used to estimate the cumulative mortality rates in the two groups. Categorical data are summarized as counts and percentage. Continuous data are summarized as mean (standard deviation) or median (25th percentile, 75th percentile).

## Results

### Cohort characteristics

**Fig. 1:**
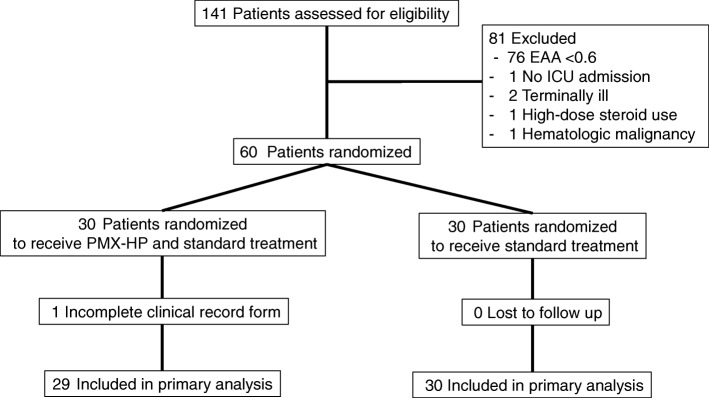
Flow diagram of patient allocation. EAA endotoxin activity assay, ICU intensive care unit, PMX-HP polymyxin B hemoperfusion

We recruited 141 severe sepsis/septic shock patients. Sixty had EAA ≥ 0.6 and were randomized equally to the PMX-HP and non-PMX-HP groups (Fig. 1). One patient from the PMX-HP group had incomplete data and was left out of the analysis. All 29 patients in the PMX-HP group received two sessions of PMX-HP. Two patients had circuit interruption, one due to technical problems and another due to circuit clotting. Baseline characteristics between the two groups were similar except for the rate of renal replacement therapy (RRT), which was higher in the PMX-HP group (93% vs 69%, *P* = 0.019) (Table 1). CRRT was the most common type of RRT in both groups (77.8% for PMX-HP group, and 70% for non-PMX-HP group), followed by sustained low-efficiency dialysis. CVVHDF was the only mode of CRRT used in the study.

**Table 1:** Baseline characteristics of treatment groups

Characteristic	PMX-HP group (*n* = 29)	Non-PMX-HP group (*n* = 30)	*P* value^a^
Age (years), mean (SD)	70.2 (13.0)	67.4 (14.6)	0.43
Male, *n* (%)	23 (79)	17 (57)	0.06
BMI (kg/m^2^), mean (SD)	24.2 (4.3)	23.5 (4.7)	0.62
Baseline serum creatinine (mg/dl), median (Q1, Q3)	1.0 (1.0, 1.1)	1.0 (0.8, 1.1)	0.48
BUN at enrollment (mg/dl), median (Q1, Q3)	41 (26, 63)	41 (22, 62)	0.65
Serum creatinine at enrollment (mg/dl), median (Q1, Q3)	2.2 (1.6, 3.1)	2.6 (1.8, 4.0)	0.34
AKI, *n* (%)			0.16
Non-AKI	4 (14)	5 (17)	
Stage 1	8 (28)	4 (13)	
Stage 2	9 (31)	5 (17)	
Stage 3	8 (28)	16 (53)	
RRT, *n* (%)	27 (93)	20 (69)	*0.02*
CRRT	21 (72%)	14 (48%)	
SLED	6 (21%)	4 (14%)	
Peritoneal dialysis	0 (0%)	2 (7%)	
SOFA score, mean (SD)	13.8 (4.4)	13.3 (5.0)	0.71
Type of fluid, *n* (%)			
Crystalloid	23 (79)	23 (77)	0.81
Colloid	15 (52)	13 (43)	0.52
Inotrope/vasopressor use*, n* (%)	25 (86)	25 (83)	> 0.99
Adrenaline	8 (28)	6 (20)	0.49
Noreadrenaline	20 (69)	22 (73)	0.71
Dopamine	8 (28)	4 (13)	0.17
Dobutamine	4 (14)	3 (10)	0.71
Vasopressin	0 (0)	0 (0)	> 0.99
Terlipressin	1 (3)	0 (0)	0.49
Inotropic score (μg/kg/min), median (Q1, Q3)	36 (7, 70)	30 (2, 83)	0.96
Vasopressor index (μg/kg/min/mmHg), median (Q1, Q3)	0.4 (0.1, 1.1)	0.4 (0.1, 1.1)	0.93
EAA at enrollment, median (Q1, Q3)	0.8 (0.7, 1.0)	0.7 (0.7, 0.9)	0.24
Presepsin (pg/ml), median (Q1, Q3)	5514 (4088, 10,513)	5030 (2459, 10,734)	0.47

Data represent sample size and column percentage unless otherwise noted

*PMX-HP* polymyxin B hemoperfusion, *SD* standard deviation, *BMI* body mass index, *Q1* 25th percentile, *Q3* 75th percentile, *BUN* blood urea nitrogen, *AKI* acute kidney injury, *RRT* renal replacement therapy, *CRRT* continuous renal replacement therapy, *SLED* sustained low-efficiency dialysis, *CVS SOFA* cardiovascular Sequential Organ Failure Assessment, *EAA* endotoxin activity assay

^a^Fisher’s exact test for categorical variables; Student’s *t* test or Kruskal–Wallis test for continuous variables, Italic data represent *P* value statistically significant

Isolated microorganisms and site of infection are presented in Table 2. Gram-negative bacteria were the predominant organisms while the respiratory tract was the most common site of infection.

**Table 2:** Isolated microorganisms by treatment group

Characteristic	PMX-HP group (*n* = 29)	Non PMX-HP group (*n* = 30)	Overall (*N* = 59)	*P* value^a^
Organism				
Gram-positive bacteria	7 (23%)	2 (7%)	9 (15%)	0.15
Gram-negative bacteria	13 (43%)	18 (60%)	31 (53%)	0.20
Fungal infection	1 (3%)	2 (7%)	3 (5%)	1.00
Mycobacterium	1 (3%)	0 (0%)	1 (2%)	1.00
Other	0 (0%)	1 (3%)	1 (2%)	1.00
Site of infection				
Central nervous system	2	0	2 (3%)	0.14
Respiratory tract	6	12	18 (31%)	0.11
Gastrointestinal tract	9	6	15 (25%)	0.33
Genitourinary tract	3	3	6 (10%)	0.97
Musculoskeletal	0	3	3 (5%)	0.08
Systemic infection	5	4	9 (15%)	0.68
Other	5	3	8 (14%)	0.42

Data represent sample size and column percentage

*PMX-HP* polymyxin B hemoperfusion

^a^Fisher’s exact test for categorical variables; Student’s *t* test or Kruskal–Wallis test for continuous variables

### Primary outcome

**Fig. 2:**
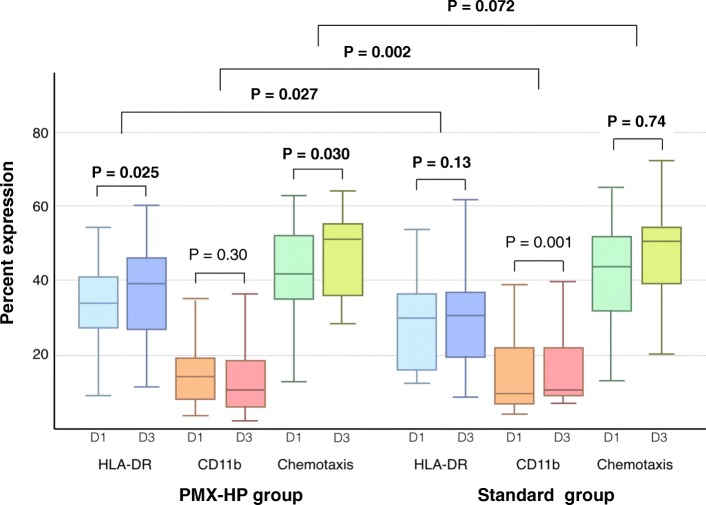
Comparison of mHLA-DR expression, CD11b expression, and chemotaxis on neutrophil between PMX-HP group and non-PMX-HP group. D day, mHLA-DR monocytic human leukocyte antigen, PMX-HP polymyxin B hemoperfusion

Baseline median mHLA-DR expression did not differ between the PMX-HP and non-PMX-HP groups. In the PMX-HP group the median mHLA-DR expression significantly increased from 33.4% (25th and 75th percentiles 27.4%, 40.7%) on day 1 to 39.2% (27.1%, 40.1%) on day 3 (*P* = 0.025) (Table 3, Fig. 2). In the non-PMX-HP group there was no change in the median mHLA-DR expression between day 1 and day 3: 30.0% (15.8%, 36.6%) vs 30.7% (19.5%, 36.8%) (*P* = 0.13). The median mHLA-DR expression on day 3 was significantly higher in the PMX-HP group than in the non PMX-HP group (*P* = 0.027). The median change in mHLA-DR expression between day 3 and day 1 was significantly higher in the PMX-HP group than in the non PMX-HP group (*P* = 0.027). At each time point, there was no significant difference of monocyte count and neutrophil count between the two groups. (Additional file 1: Table S1).

**Table 3:** Physiological end points by treatment group

Outcome	PMX-HP group			Non-PMX-HP group				
	Baseline (*n* = 29)	Day 3 (*n* = 26)	*P* value within group^a^	Baseline (*n* = 30)	Day 3 (*n* = 20)	*P* value within group^b^	*P* value between groups on day 3^c^	*P* value between group change from baseline and day 3^d^
Primary outcome								
mHLA-DR expression (%), median (Q1, Q3)	33.4 (27.4–40.7)	39.2 (27.1–45.8)	*0.025* ^**e**^	30 (15.8–36.6)	30.7 (19.5–36.8)	0.13	*0.027 (0.042)* ^**e**^	*0.027* (0.11)^**e**^
mHLA-DR expression (MFI), mean (SD)	481.4 (131.5)	509.0 (139.9)	0.78	446.6 (142.1)	447.3 (148.9)	0.35	0.16 *(0.035)*^**e**^	0.34 *(0.038)*^**e**^
Secondary outcomes								
CD11b (%), mean (SD)	15.4 (9)	13.7 (9.5)	0.30	14.3 (10.5)	16.2 (10.1)	*0.001*	0.39 (0.15)^**e**^	*0.002 (0.001)* ^**e**^
CD-11b (MFI), mean (SD)	189.7 (57.1)	165.4 (53.1)	*0.045*	186.2 (53.3)	223.5 (48.0)	*< 0.001*	*0.001 (< 0.001)* ^**e**^	*< 0.001 (< 0.001)* ^**e**^
Neutrophil chemotaxis (%), mean (SD)	42.8 (12.5)	47.9 (10.2)	*0.030*	42.3 (13.7)	46.2 (12.3)	0.74	0.60 (0.77)^**e**^	0.07 (0.08)^**e**^
Presepsin (pg/ml), median (Q1, Q3)	5514 (4088, 10,513)	2895 (2190, 6038)	0.059	5030 (2459, 10,734)	3468 (2204, 6038)	0.12	0.93	0.38
CVS SOFA score, median (Q1, Q3)	4 (4, 4)	3 (0, 4)	*0.003*	4 (4, 4)	3 (1, 4)	0.26	0.71	0.16
Inotropic score, median (Q1, Q3)	36 (6.6, 70)	11 (1, 49.2)	0.13	30 (2, 83)	14 (0, 36)	0.94	0.57	0.20
Vasopressor dependency index (mmHg^−1^), median (Q1, Q3)	0.4 (0.1, 1.1)	0.1 (0.01, 0.7)	0.14	0.4 (0.1, 1.1)	0.2 (0, 0.6)	0.96	0.60	0.25
Noradrenaline (μg/kg/min), median (Q1, Q3)	0.2 (0.0, 0.4)	0.01 (0, 0.2)	0.23	0.3 (0.0, 0.4)	0.1 (0, 0.4)	0.85	0.42	0.42
EAA level, median (Q1, Q3)	0.8 (0.7, 1)	0.7 (0.6, 0.9)	0.11	0.7 (0.7, 0.9)	0.6 (0.5, 0.9)	0.14	0.84	0.90

Data represent median (25th percentile, 75th percentile) unless otherwise noted

*PMX-HP* polymyxin B hemoperfusion, *mHLA-DR* monocyte human leukocyte antigen, *Q1* 25th percentile, *Q3* 75th percentile, *MFI* mean fluorescence intensity, *SD* standard deviation, *CVS SOFA* cardiovascular Sequential Organ Failure Assessment, *EAA* endotoxin activity assay, Italic data represent *P* value statistically significant

^a^*P* value for comparison between day 1 and day 3 within PMX-HP group using Student’s *t* test or Kruskal–Wallis test

^b^*P* value for comparison between day 1 and day 3 within non-PMX-HP group using Student’s *t* test or Kruskal–Wallis test

^c^*P* value for comparison between PMX-HP and non-PMX-HP groups on day 3 using Student’s *t* test or Kruskal–Wallis test

^d^*P* value for comparing groups on mean or median change between day 3 and day 1 using Student’s *t* test or Kruskal–Wallis test

^**e**^Adjusted for renal replacement therapy status

### Secondary outcomes

In the PMX-HP group, the mean CD11b expression on neutrophils did not significantly change between days 1 and 3 (from 15.4% to 13.7%, *P* = 0.30), while in the non-PMX-HP group it significantly increased from day 1 to day 3 (from 14.3% to 16.2%, *P* = 0.001). The mean change in CD11b between day 3 and day 1 was significantly lower in the PMX-HP group than in the non-PMX-HP group (*P* = 0.002) (Table 3, Fig. 2).

In the PMX-HP group, the mean neutrophil chemotaxis significantly increased from day 1 to day 3 (from 42.8% to 47.9%, *P* = 0.03), while in the non-PMX-HP group there was no change (from 42.3% to 46.2%, *P* = 0.73). However, we did not detect a difference between groups in the mean change from baseline (*P* = 0.07). There was improvement in the CVS SOFA score in the PMX-HP group (*P* = 0.003) but not in the non-PMX-HP group (*P* = 0.26). However, we did not detect a difference between groups in the median change from baseline (*P* = 0.16).

We did not notice a difference between groups in the median change from baseline in presepsin, inotropic score, vasopressor dose, or noradrenaline dose. There was no significant change in EAA levels from baseline within or between groups. We also did not see a difference between groups in mortality (days 7 or 28), ICU-free days, ventilator-free days, dialysis dependence status, renal recovery, serum creatinine on day 28, vasopressor-free days, and MAKE 28 (Table 4).

**Table 4:** Clinical outcomes by treatment group

	PMX-HP group	Non PMX-HP group	*P* value^a^
Overall	*n* = 29	*n* = 30	
Mortality on day 3, *n* (%)	3 (10.3)	10 (33.3)	0.058
Mortality on day 7, *n* (%)	6 (20.7)	12 (40.0)	0.11
Mortality on day 28, *n* (%)	17 (58.6)	15 (50.0)	0.51
MAKE 28^b^ (%)	22 (75.9)	22 (73.3)	1.00
Alive on day 28	n=13^c^	*n* = 15	
ICU-free days (Q1, Q3)	0 (0, 10)	0 (0, 8)	0.99
Ventilator-free days (Q1, Q3)	0 (0, 8)	0 (0, 0)	0.28
RRT, *n* (%)	7 (53.8)	6^d^ (50.0)	0.85
Serum creatinine (mg/dl) (Q1, Q3)	1.4 (1.1, 2.2)	2.1 (1.3, 2.5)	0.59
Vasopressor free days (Q1, Q3)	0 (0, 25)	11.5 (0, 25)	0.47
AKI at baseline	*n* = 25	*n* = 25	
Renal recovery^e^, *n* (%)	5 (20.0)	3 (12.0)	0.70

Data represent median (25th percentile, 75th percentile) unless otherwise noted

Data measured on day 28 unless otherwise noted

*PMX-HP* polymyxin B hemoperfusion, *ICU* intensive care unit, *Q1* 25th percentile, *Q3* 75th percentile, *RRT* renal replacement therapy, *AKI* acute kidney injury

^a^Fisher’s exact test for categorical variables; Kruskal–Wallis test for continuous variables

^b^Major adverse kidney events on day 28, comprised of death, or dialysis dependence, or persistent AKI on day 28

^c^In the PMX-HP group one patient died by the end of day 28, but his values were measured and used in the analysis

^d^In the non-PMX-HP group, RRT data were available in 12 patients

^e^Renal recovery mean alive, dialysis independence and no AKI on day 28 after enrollment

### Adverse events

During the study there were no adverse events due to bleeding or hypo/hypertension, and no events indicative of neurotoxicity or nephrotoxicity related to polymyxin B were reported.

## Discussion

### Key findings

The main finding of our study is that in patients with severe sepsis after PMX-HP treatment, mHLA-DR expression increased and CD11b expression decreased compared to standard care. Also, in patients treated with PMX-HP there was a significant increase in neutrophil chemotaxis and a decrease in CVS SOFA score between days 1 and 3 (Table 3). Due to our small sample size, we could not calculate the adjusted impact of PMX-HP on 28-day mortality. However, in univariate analysis, we found no significant difference in mortality rates between the PMX-HP and non-PMX-HP groups (Table 4, Additional file 1: Figure S1). We did not encounter any safety issues using PMX-HP. Other secondary outcomes including ICU-free days, mechanical ventilator-free days, RRT, renal recovery, vasopressor-free days, and MAKE 28 were not significantly different between treatment groups on day 28 (Table 3).

The improvement in mHLA-DR expression and neutrophil chemotaxis activity (only in the PMX-HP group) after PMX-HP correspond to the “Cytokinetic Theory”, one of the new concepts of blood purification, proposed by Peng and by Kellum et al. [27]. This theory suggested that blood purification improved “leukocyte reprogramming” by acting at the cellular level of monocyte and neutrophil function. Data from uncontrolled clinical studies supported the “Cytokinetic Theory” [13, 28]. However, the mechanism of PMX-HP in improving mHLA-DR expression is still unclear. We hypothesized that PMX-HP reduced circulating endotoxin, which is one of the major drivers of intravascular inflammation. This may have led to a reduced intense inflammatory response/cytokine burden in the blood and subsequently to two separate effects—attenuated immunosuppression and restoration of chemokine gradients between the circulation and the site of infection. Although our study could not demonstrate the difference of EAA level on day 3 after treatment (Table 3), there are some possible explanations. First, the production rate of endotoxin might have been higher than the removal rate of endotoxin by PMX-HP. Secondly, the dose of PMX-HP (2 h/session, 2 days) might not have been enough in some septic patients who had a high endotoxin burden.

Neutrophils play an important role in the inflammatory response in sepsis [5]. Impaired neutrophil function is also associated with subsequent nosocomial infection [29]. The improvement in neutrophil chemotaxis in our study could be explained by the “Cytokinetic Theory”. After the direct effect of removing endotoxins and the indirect effect of removing inflammatory mediators from the blood compartment by PMX-HP there might be an increase in the cytokine/chemokine concentration gradient from plasma to infected tissue. Consequently, neutrophils could move toward the site of infection and allow an increase in local bacterial clearance [28]. The results from our study indicate that severe sepsis patients, who still suffer from an immunoparalysis state as demonstrated by low mHLA-DR expression or impaired chemotaxis function, might receive benefits from PMX-HP treatment.

CD11b is constitutively present on neutrophils. CD11b with CD18 as the CD11b/18 complex is composed of three heterodimers, each having a common subunit noncovalently linked to a different subunit, CD11a, CD11b, and CD11c, and its main activity is the regulation of the adhesive interactions of cells with other cells and with the extracellular matrix. CD11b is rapidly upregulated in response to inflammation and proinflammatory cytokines, and thus considered to represent neutrophil activation [30, 31]. Our study showed that neutrophil activation was stabilized in the PMX-HP group compared to the non-PMX-HP group (Fig. 2, Table 3, *P* = 0.002).

Two recently published RCTs examining the efficacy of PMX-HP reached different conclusions. The Early Use of Polymyxin B Hemoperfusion in Abdominal Septic Shock (EUPHAS) [32], a single-center RCT in Italy, investigated the role of PMX-HP in severe sepsis/septic shock post abdominal surgery and showed that mortality at 28 days was significantly lower in the PMX-HP group than in the standard treatment group. Conversely, a multicenter trial in France (ABDOMIX study) investigated the role of PMX-HP in the same group of patients and could not find a benefit of PMX-HP on mortality [33]. Of note, there were only 81 of 119 patients (69.8%) who received two PMX-HP sessions in the ABDOMIX study. Both studies did not use the EAA level to guide PMX-HP initiation. The latest meta-analysis by Fujii et al. [34] included six trials and 857 participants, and could not show the benefit of PMX-HP on 28-day mortality and organ dysfunction scores. Again, most of the included studies did not use the endotoxin level to guide intervention.

### Strengths and limitations

Our study has several strengths. First, this study is the first RCT to explore the role of PMX-HP in improvement of monocyte (defined by mHLA-DR expression) and neutrophil (defined by neutrophil chemotaxis, and CD11b expression) function even though this therapy has been available for more than 25 years. Secondly, we used the EAA to select patients who might receive benefits from PMX-HP. Thirdly, the second PMX-HP session was completed in 90% of participants, which allowed us to fully test the cellular effect of PMX-HP treatment. Finally, our results support the feasibility and safety of this approach as the basis for a future definitive trial. Currently, there is the largest multicenter randomized trial, Evaluating the Use of Polymyxin B Hemoperfusion in a Randomized controlled trial of Adults Treated for Endotoxemia and Septic shock (EUPHRATES), which finished in 2017 but is as yet unpublished. This study aimed to prove the concept of using a biomarker, EAA, to identify the patients who will receive the most benefit from PMX-HP treatment [35].

There are several limitations to our study. First, due to the nature of the study, this single-center study was an unblinded RCT. However, the robust standard protocol for sepsis treatment and the high compliance rates minimize the risk of bias in sepsis treatment. Secondly, the number of participants was rather small (30 in each group), possibly leading to insufficient power for detecting differences in secondary endpoints. Thirdly, the rate of RRT was higher in the PMX-HP group which could affect physiological endpoints. However, even with the imbalance in RRT rate, mHLA-DR expression increased in the PMX-HP group compared to the non-PMX-HP group. Moreover, we performed further analysis using a general linear model (GLM) to model the outcome variable in log scale adjusting for RRT status, and mHLA-DR on day 3 in PMX-HP patients was still significantly higher than in non-PMX-HP patients (38.3% vs 30.2%, *P* = 0.042) (Table 1). Lastly, although EAA was considered the only clinically available technique to detect endotoxin activity, the lack of a quantitative technique for endotoxin levels in humans continues to be problematic. EAA, a ratio of activity, has not been validated versus a quantitative measurement in a large population of septic patients. However, with the current evidence, this EAA seems to be a promising method. EAA has shown a relationship with lipopolysaccharide (LPS) concentration [17, 36]. LPS is well known to cause an inflammatory response and organ dysfunction. Moreover, EAA can reflect the severity of sepsis and correlates with clinical outcome [19, 37, 38]. Lastly, EAA is approved by the US FDA and received a CE marked assay.

## Conclusions

PMX-HP showed the potential to improve immunoparalysis status in severe sepsis/septic shock patients and should be considered as a potential treatment strategy for this specific setting.

## Additional file

Additional file 1: **Table S1.** Monocyte and neutrophil counts stratified by treatment group. **Figure S1.** Survival curves for PMX-HP and non-PMX-HP groups (blue line, PMX-HP; red line, non-PMX-HP). Kaplan–Meier curve for probability of survival to day 28. PMX-HP polymyxin B hemoperfusion. (DOCX 39 kb)

## Abbreviations

CVS: Cardiovascular
EAA: Endotoxin activity assay
FMLP: Formyl methionyl-phenylalanine
MHC: Major histocompatibility complex
mHLA-DR: Monocytic human leukocyte antigen
ICU: Intensive care unit
LPS: Lipopolysaccharide
PMN: Polymorphonuclear cells
PMX-HP: Polymyxin B hemoperfusion
ROS: Reactive oxygen species
RRT: Renal replacement therapy
SOFA: Sequential Organ Failure Assessment
