# Supplementary material for: The effect of polymyxin B hemoperfusion on modulation of human leukocyte antigen DR in severe sepsis patients
Source: Crit Care. 2018 Oct 26;22:279. doi: 10.1186/s13054-018-2077-y (PMC6204024; doi:10.1186/s13054-018-2077-y)
Supplement: Supplementary file 1 — Table S1. Monocyte and neutrophil counts stratified by treatment group. Figure S1. Survival curves for PMX-HP and non-PMX-HP groups (blue line, PMX-HP; red line, non-PMX-HP). Kaplan–Meier curve for probability of survival to day 28. PMX-HP polymyxin B hemoperfusion. (DOCX 39 kb) [file 13054_2018_2077_MOESM1_ESM.docx]

**Additional file 1**

**Table S1.** Number of Monocytes and Neutrophils count stratified by treatment group

| **Outcomes** | **PMX-HP Group** | | | **Non PMX-HP Group** | | | | |
| --- | --- | --- | --- | --- | --- | --- | --- | --- |
|  | **Day 1 (N=29)** | **Day 3 (N=26)** | **P-value within group** | **Day 1 (N=30)** | **Day 3 (N=20)** | **P-value within group** | **P-value**  **between group**  **on Day 3** | **P-value**  **between group, difference**  **Day1-3** |
| Monocyte, median (Q1,Q3)* | 4.4  (3.0, 7.4) | 4.8  (3.6, 7.6) | 0.25 | 5.3  (2.9, 6.5) | 3.5  (2.0, 5.1) | 0.79 | 0.06 | 0.28 |
| Neutrophil, median (Q1,Q3)* | 7.5  (2.9, 9.4) | 7.7  (4.9, 11.2) | 0.74 | 7.7  (4.6, 12.1) | 5.0  (3.0, 10.0) | 0.41 | 0.58 | 0.65 |

* unit in log scale

**Figure S1.** Survival curves for PMX-HP and non PMX-HP groups (blue line, PMX-HP; red line, non PMX-HP). The figure shows Kaplan-Meier curve for the probability of survival to day 28

PMX-HP; Polymyxin-B Hemoperfusion

P = 0.72
